# A Long Time Constant May Endorse Sharp Waves and Spikes Over Sharp Transients in Scalp Electroencephalography: A Comparison of After-Slow Among Different Time Constants Concordant With High-Frequency Activity Analysis

**DOI:** 10.3389/fnhum.2021.748893

**Published:** 2021-10-22

**Authors:** Shamima Sultana, Takefumi Hitomi, Masako Daifu Kobayashi, Akihiro Shimotake, Masao Matsuhashi, Ryosuke Takahashi, Akio Ikeda

**Affiliations:** ^1^Department of Neurology, Graduate School of Medicine, Kyoto University, Kyoto, Japan; ^2^Department of Clinical Laboratory Medicine, Graduate School of Medicine, Kyoto University, Kyoto, Japan; ^3^Department of Epilepsy, Movement Disorders and Physiology, Graduate School of Medicine, Kyoto University, Kyoto, Japan

**Keywords:** epileptiform discharge, total area, total duration, paroxysmal depolarization shifts, high-frequency activity

## Abstract

**Objective:** To clarify whether long time constant (TC) is useful for detecting the after-slow activity of epileptiform discharges (EDs): sharp waves and spikes and for differentiating EDs from sharp transients (Sts).

**Methods:** We employed 68 after-slow activities preceded by 32 EDs (26 sharp waves and six spikes) and 36 Sts from 52 patients with partial and generalized epilepsy (22 men, 30 women; mean age 39.08 ± 13.13 years) defined by visual inspection. High-frequency activity (HFA) associated with the apical component of EDs and Sts was also investigated to endorse two groups. After separating nine Sts that were labeled by visual inspection but did not fulfill the amplitude criteria for after-slow of Sts, 59 activities (32 EDs and 27 Sts) were analyzed about the total area of after-slow under three TCs (long: 2 s; conventional: 0.3 s; and short: 0.1 s).

**Results:** Compared to Sts, HFA was found significantly more with the apical component of EDs (*p* < 0.05). The total area of after-slow in all 32 EDs under TC 2 s was significantly larger than those under TC 0.3 s and 0.1 s (*p* < 0.001). Conversely, no significant differences were observed in the same parameter of 27 Sts among the three different TCs. Regarding separated nine Sts, the total area of after-slow showed a similar tendency to that of 27 Sts under three different TCs.

**Significance:** These results suggest that long TC could be useful for selectively endorsing after-slow of EDs and differentiating EDs from Sts. These findings are concordant with the results of the HFA analysis. Visual inspection is also equally good as the total area of after-slow analysis.

## Key Points

– Precise detection of EDs is important for patients with epilepsy.
– The long TC setting is appropriate to augment the after-slow activity of EDs.
– The findings may endorse the operational definitions of EDs and Sts.
– These findings are also consistent with the analysis of HFA.

## Introduction

Electroencephalography (EEG) is a useful clinical examination for the diagnosis of epilepsy since it is the only clinically available examination to delineate the paroxysmal depolarization shifts (PDS). Appropriate EEG readings, especially for the detection of epileptiform discharges (EDs), are crucial for making the diagnosis of epilepsy (Winesett and Benbadis, 2008; Gil-Nagel and Abou-Khalil, 2012). According to the guidelines of the International Federation of Clinical Neurophysiology (IFCN), EDs are defined as transient activity that is outstanding from background activity with a pointed peak at a conventional paper speed or time scale, mainly negative component, and durations from 20 to less than 70 ms for spikes and from 70 to 200 ms for sharp waves (Brazier et al., 1961; Noachtar et al., 1999). However, it is sometimes still inaccurate even for certified electroencephalographers to distinguish EDs from morphologically resembling normal or non-specific sharply contoured waveforms, namely, sharp transients (Sts) (Janca et al., 2014). On the other hand, EDs are usually accompanied by a subsequent slow wave referred to as an “after-slow wave,” which is helpful to identify EDs and to aid such differentiation (Winesett and Benbadis, 2008; Selwa, 2010). According to the study by Aykut et al. (2020), they found that three criteria: waves with spiky morphology, followed by an after-slow wave and voltage map suggesting a source in the brain, providing the best diagnostic value with specificity over 95% (Aykut et al., 2020). However, this study did not investigate the degree of after-slow activity to define EDs.

Clinical EEG technology has developed over the past several decades. Digital EEG has become common because of the advantages of recording, reviewing, and storing EEG data (Nuwer, 1997). Thus, current digital EEG data are recorded, analyzed, and interpreted by using a wide range of time constant (TC), as clinically needed (Maus et al., 2011; Constantino and Rodin, 2012). If long TC is chosen (e.g., 2 s), slow activities are not deleted as compared with the conventional settings (Fisch and Spehlmann, 1999; Sinha et al., 2016). This may be beneficial for detecting slow EEG activities (Misra and Kalita, 2005), including the after-slow activity of EDs. However, the degree of detection of the after-slow activity of EDs under different TC conditions has not been systematically analyzed. To the best of our knowledge, there was no study about the endorsement of the definition of Sts with less after-slow as compared with EDs.

In this study at first, we investigate whether long TC is useful for detecting the after-slow activity of EDs and for differentiating EDs from Sts as compared with conventional TC settings and whether the current operational definition of EDs and Sts is appropriate. The target activities must be visually identified and selected “typical” patterns inspected by EEG experts. It is because, despite recent advances in information technology, EEG interpretation still requires trained EEG experts to define clinically useful specific EEG results, and then we tried to endorse the EEG experts’ judge by means of parameter analysis of waveform analysis. Second, recent technological advancement of digital EEG has aided the analysis of high-frequency activity (HFA) even in the scalp EEG (Kobayashi et al., 2004; Shibata et al., 2016). As HFA is considered to have an association with epileptogenicity (Shibata et al., 2016), we also examine the relationship of HFA with EDs and Sts for the endorsement of these two groups. Once those two approaches of this study would provide positive results, we may skip complicated HFA analysis, and only simple visual analysis by means of long TC may be practically useful.

## Materials and Methods

### Study Design and Participants

We retrospectively analyzed randomly selected 55 routine EEGs from 52 patients (22 men, 30 women; mean age ± SD, 39.08 ± 13.13 years; age range, 18–80 years) with partial and generalized epilepsy. All EEGs were recorded at the Kyoto University Hospital from June 2013 to July 2017. Among the 52 epilepsy patients, 37 were clinically diagnosed as having partial epilepsy (temporal = 12, frontal = 13, parietal = 3, and other lobes or in combination = 9) and 15 as having generalized epilepsy (idiopathic generalized epilepsy = 6, symptomatic generalized epilepsy = 5, genetic epilepsy with febrile seizure plus = 1, and other generalized epilepsy = 3). We included both partial and generalized epilepsy since we tried to extract the common feature of EDs and Sts regardless of epilepsy classification. The study protocol was approved by the Institutional Review Board of the Kyoto University (No. R0603).

### Electroencephalography Recording

Conventional digital EEGs by using the scalp electrodes were recorded for at least 30 min (EEG-1100 Neurofax; Nihon Kohden, Tokyo, Japan). Conventional Ag/AgCl scalp electrodes with a diameter of 10 mm were used. The Fp1, Fp2, Fz, F3, F4, F7, F8, Cz, C3, C4, T3, T4, Pz, P3, P4, T5, T6, O1, O2, A1, and A2 electrodes were placed according to the International 10–20 system. In addition, T1 and T2 electrodes (Silverman, 1965) were placed in 12 EEGs. The EEG data were recorded with a sampling rate of 500 Hz, the high-frequency filter of 120 Hz, and TC of 2 s (13 EEGs) or 10 s (42 EEGs). The impedance of all the electrodes was kept below 5 kΩ. EEG was reviewed and the EEG report was made by the certified electroencephalographer.

### Selection of Electroencephalography Activities

First, we reviewed the EEGs with the sensitivity of 10 μV, TC of 0.3 s (regardless of the recording TC condition of EEGs), and a high-frequency filter of 120 Hz and averaged reference montage of all the electrodes by using the review software of the Nihon Kohden. Second, we randomly selected the typical samples (for EDs: clear apical component with large after-slow; for Sts: clear apical component with small after-slow) of 68 EEG activities according to annotations that had been already labeled only for the clinical purposes through visual inspection by the certified electroencephalographers to avoid the selection biases. Because of these reasons, the adopted number of samples is limited since we respected the initial impression and decision of electroencephalographers. The 68 visually defined EEG samples consisted of 32 EDs [26 sharp waves (all focal), six spikes (5: focal and 1: generalized)], and 36 Sts (all focal). Third, we adopted the criteria used for the EDs (sharp waves and spikes) as follows: (1) standing out from the background, (2) the amplitude of after-slow component being > 50% of the amplitude of the immediately preceding apical component, and (3) total duration of apical component up to 200 ms. Then, we differentiated sharp waves and spikes based on the total duration of the apical component (spikes: < 70 ms and sharp waves: 70–200 ms).

**FIGURE 1:**
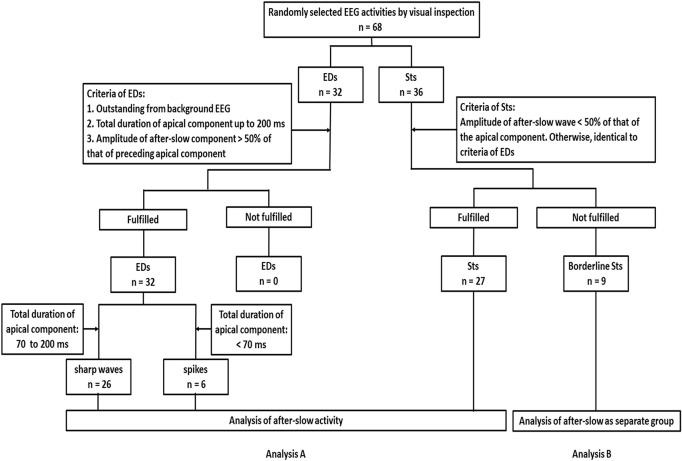
Flow diagram of recruitment and classification of epileptiform discharges [epileptiform discharges (EDs): sharp waves and spikes] and sharp transients (Sts) based on the criteria of this study. First, a random selection of typical samples of 68 Electroencephalography (EEG) activities is done according to the annotations that have been already labeled for clinical purposes by visual inspection. The 68 EEG activities consist of 32 EDs and 36 Sts. Then the following criteria are adopted for EDs: (1) outstanding from the background EEG, (2) total duration of apical component up to 200 ms, and (3) the amplitude of after-slow component being > 50% of the amplitude of the immediately preceding apical component. About the criteria for the Sts, the amplitude of the after-slow component has to be < 50% that of the apical component. Otherwise, identical to criteria of EDs. Analysis A, according to these amplitude-quantitative criteria, among 68 activities, 32 EDs and 27 Sts fulfill the criteria. Then sharp waves and spikes are differentiated based on the total duration of the apical component (sharp waves: 70–200 ms and spikes: < 70 ms) and analysis of after-slow activities is performed. Analysis B, the remaining nine Sts do not fulfill the amplitude criteria but are visually defined, so that they are labeled as borderline Sts. They are considered and analyzed as a separate group.

With respect to the criteria for the Sts, only the difference from the criteria of EDs was that the amplitude of the after-slow component had to be < 50% compared to the apical component. According to these amplitude-quantitative criteria, 59 samples (32 EDs and 27 Sts) out of 68 samples (32 EDs and 36 Sts) fulfilled these criteria (Analysis A in Figure 1). On the other hand, the remaining nine Sts showed that the amplitude of the after-slow component was > 50% compared to the apical component and, thus, only compatible as visually defined samples. They were labeled as “borderline Sts” and separately analyzed (Analysis B in Figure 1).

The definition of after-slow activity was as follows: slow activity immediately following the apical component and the similar distribution and the same polarity as the preceding apical component (Selwa, 2010).

### Analysis of Electroencephalography Activities

After selecting the 59 EEG samples, the 5 s EEG segments including the selected activities were chosen, and the following analysis was performed by using averaged reference montage of all the electrodes (MATLAB version R2015b; MathWorks, Natick, MA, United States). We visually selected one channel with the maximum apical component. In the case of the contaminated artifacts, no clear onset or end of either apical or after-slow component or the absence of an outstanding after-slow wave, then the second maximum apical activity in a different channel was selected (eight activities). For analysis with different TC, we adopted three different TC conditions (long TC: 2 s; conventional TC: 0.3 s; and short TC: 0.1 s).

**FIGURE 2:**
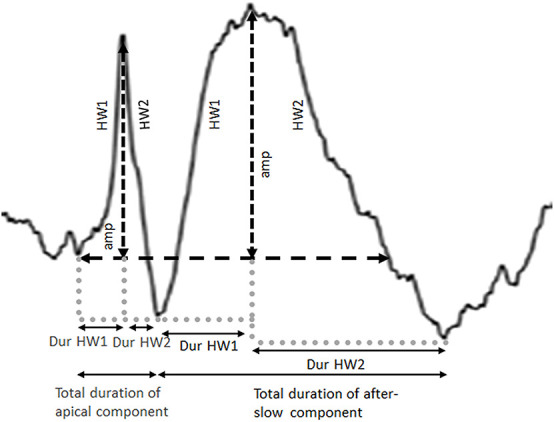
Definition of amplitude (amp) and total duration calculated for both the apical and after-slow wave components. Each apical and after-slow wave component is divided into two segments, half-wave 1 (HW1: the ascending phase) and half-wave 2 (HW2: the descending phase), by the peak detection algorithm, and the amplitudes of the apical and after-slow wave components are calculated. The duration of each half-wave (Dur HW1 and Dur HW2) is calculated by defining the time between the corresponding minima and peak.

After selecting the channel, the apical and after-slow components were divided into two segments—half-wave 1 (HW1; the ascending phase) and half-wave 2 (HW2; the descending phase) —by the peak detection algorithm (Figure 2). Next, the amplitudes of the apical and after-slow components were calculated by taking the onset of the apical component as the baseline point (Figure 2). The duration of each HW1 and HW2 was measured by defining the time between the corresponding minima and peak. The total duration of the apical and after-slow components was calculated as the sum of the durations of HW1 and HW2, respectively (Figure 2; Dingle et al., 1993; Rakhade et al., 2007).

**FIGURE 3:**
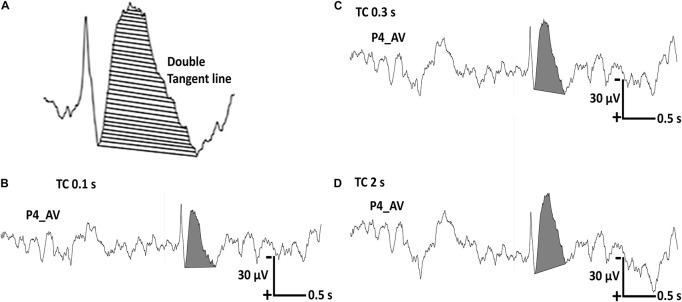
Representative regional sharp wave in one patient with a recording time constant (TC) of 10 s. **(A)** The total area of the after-slow is calculated using the double tangent line method. **(B)** The total area of the after-slow under TC 0.1 s is 9.6837 μVs. **(C,D)** The same parameter under TC 0.3 s is 13.8499 μVs and that under TC 2 s is 15.6579 μVs, respectively.

About the analysis of after-slow activity, i.e., amplitude vs. total area, we finally adopted only the total area as an analysis parameter under the three different TCs by using the double tangent line method (Figure 3A). We did not adopt the amplitude because the baseline point of background activity for amplitude measurement varied very much when the display TC was changed.

### Analysis of Borderline Sharp Transients

About nine Sts that did not fulfill the amplitude criteria of Sts were labeled as “borderline Sts” (Analysis B in Figure 1). Similar to Analysis A in Figure 1, the total area of the after-slow activities was analyzed under the three different TCs.

### Analysis of High-Frequency Activity

For further endorsement of two groups of EDs and Sts, HFA associated with the apical component of the EDs and Sts was also investigated (MATLAB version R2017b; MathWorks, Natick, MA, United States). Before analysis of HFA for appropriate averaged montage, if the EEG signal of a certain electrode was found to be accompanied by any artifact, the electrode channel with artifact was excluded from the reference channels of the averaged montage (8 EDs and 15 Sts). We tested two frequency bands, one with a low-frequency filter with 40 Hz and the other with an 80 Hz, zero-phase bidirectional fourth-order Butterworth filter. HFA was defined as an event of at least two consecutive peaks in these frequency bands, amplitude greater than -2 SD from the baseline, interval less than 0.03 and 0.015 s for 40 and 80 Hz, respectively. HFA was examined within the period of 50 ms before and 50 ms after the visually defined peak of the apical component. If HFA (40 or 80 Hz or both) was found within or some overlapping with this period, HFA was considered as associated with the apical component of EDs and Sts.

### Statistical Analysis

To evaluate the differences in the total area of the after-slow activity among the three different TCs, we conducted the Friedman test followed by the Dunn–Bonferroni *post hoc* tests for pair wise comparison of EDs and Sts separately. We also compared the total area and total duration of the after-slow activity between EDs and Sts under the TC 0.3 s condition by using the Mann–Whitney *U*-test.

To examine the relationship between the width of apical component and size of after-slow, Kendall’s tau-b correlation analysis with scatter plots was done between the total area of after-slow of EDs and Sts under three different TCs and a total duration of apical component (calculated in TC 0.3 s), separately.

To assess the difference of HFA between EDs and Sts, we performed Pearson’s chi-squared analysis of all 68 activities (32 EDs and 36 Sts). It was also checked for 59 activities (32 EDs and 27 Sts) after excluding borderline nine Sts from 68 samples, separately. *p* < 0.05 was considered as statistically significant.

## Results

### Analysis of Total Area of After-Slow Among the Three Different Time Constant Conditions

**FIGURE 4:**
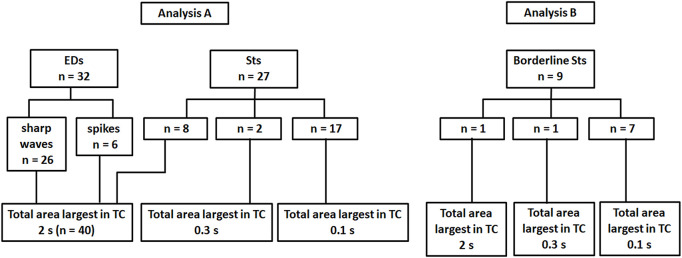
The total area of after-slow activities of 32 EDs and 36 Sts under three different TC conditions. Analysis A, the after-slow activities of all 32 EDs (26 sharp waves and six spikes) show the largest total area under TC 2 s. Among 27 Sts, eight, two, and 17 after-slow activities show the largest total area under TCs 2, 0.3, and 0.1 s, respectively. Analysis B, about the after-slow activities of nine borderline Sts, one, one, and seven activities show the largest total area under TCs 2, 0.3, and 0.1 s, respectively.

Regarding the total area of 59 after-slow, 40 activities (preceded by 26 sharp waves, six spikes, and eight Sts) showed the largest total area under TC 2s (Figures 3B–D and Analysis A in Figure 4). On the other hand, two and 17 after-slow activities (all preceded by Sts) showed the largest total area under TCs 0.3 and 0.1 s, respectively (Analysis A in Figure 4).

**FIGURE 5:**
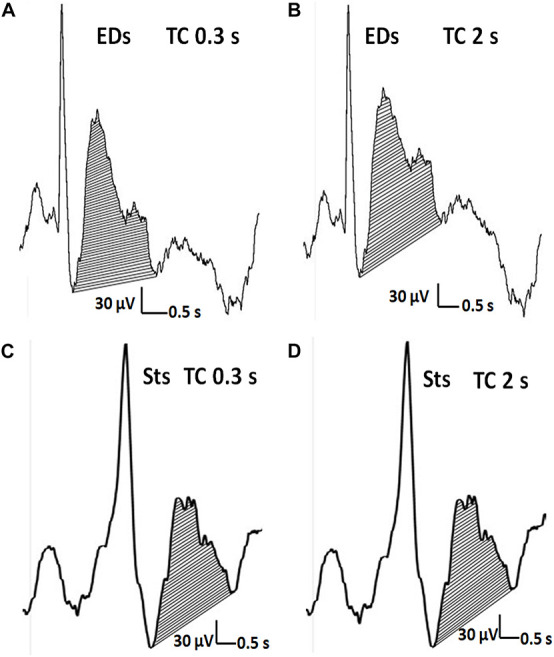
The distinction of EDs from Sts. **(A,C)** The after-slow wave of an EDs (>50% of the amplitude of preceding activity) and Sts (<50% of the amplitude of preceding activity). **(A,B)** Concerning EDs, the after-slow wave is exaggerated with TC of 2 s compared with TC of 0.3 s. **(C,D)** In contrast, the much lesser after-slow wave of Sts is almost the same as one with TC of 2 s.

**FIGURE 6:**
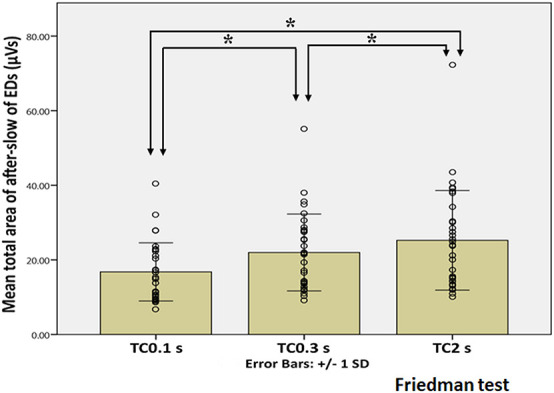
Statistical analysis of the total area of the after-slow of EDs under three different TC conditions. X and Y axes show three different TCs and the mean total area of the after-slow of EDs, respectively, error bars indicate ± 1 SD and the circle shows the total area of each EDs in three different TCs. The total area of the after-slow under TC 2 s is significantly larger than those under TC 0.3 s and 0.1 s (*p* < 0.001). The same parameter is also significantly larger under TC 0.3 s than under TC 0.1 s (*p* < 0.001). *Indicates statistical significance.

**FIGURE 7:**
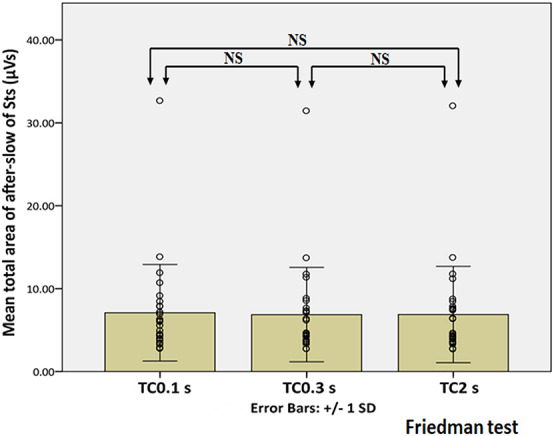
Statistical analysis of the total area of the after-slow of Sts under three different TCs. X and Y axes show three different TCs and the mean total area of the after-slow of Sts, respectively, error bars indicate ± 1 SD, and the circle shows the total area of each Sts in three different TCs. No significant difference is observed under three different TCs. NS indicates not statistically significant.

Figure 5 showed the representative waveforms of the after-slow of EDs and Sts in different TCs. It was visually evident that compared with Sts, the after-slow of EDs displayed with TC 2 s was exaggerated than that with TC 0.3 s which can be helpful to differentiate EDs and Sts visually. As for EDs, after Bonferroni adjustments, the total area of the after-slow was significantly larger under TC 2 s (mean ± SD: 25.25 ± 13.35 μVs) than under 0.3 s (21.98 ± 10.30 μVs) (*p* < 0.001) and 0.1 s (16.78 ± 7.79 μVs) (*p* < 0.001), and it was significantly larger under TC 0.3 s than that under TC 0.1 s (*p* < 0.001) (Figure 6). By contrast, no significant differences were seen in the same parameter of Sts among three different TCs (TC 2 s: 6.88 ± 5.81 μVs, TC 0.3 s: 6.87 ± 5.69 μVs, and TC 0.1 s: 7.09 ± 5.82 μVs) (*p* > 0.05) (Figure 7).

### Analysis of Total Area and Total Duration of After-Slow With Time Constant of 0.3 s

The EDs showed a significantly larger total area of the after-slow (21.98 ± 10.30 μVs) as compared with those of Sts (6.87 ± 5.69 μVs) under TC 0.3 s (*p* < 0.001) according to their definition. In addition, the total duration of the after-slow of EDs (437.19 ± 125.07 ms) was also significantly longer as compared with Sts (285.41 ± 144.21 ms) (*p* < 0.001).

With respect to the correlation analysis, no significant relationship was found in any combination between total area of after-slow and total duration of apical component in EDs (TC 2 s: τb = 0.219; TC 0.3 s: τb = 0.223 and TC 0.1 s: τb = 0.235) and Sts (TC 2 s: τb = –0.191; TC 0.3 s: τb = –0.129 and TC 0.1 s: τb = –0.077), respectively, as shown by scatter plots in Supplementary Figures 1, 2.

It was initially concerned that the apical component of EDs may have a longer duration, and thus, they may be accompanied by larger after-slow, but it did not happen. Neither happened in the case of Sts. It simply convinced us that visually defined EDs and Sts could have a similar range of duration of apical components between each other and the degree of after-slow is independent of the apical components *per se.*

### Analysis of Borderline Sharp Transients

About the nine after-slow activities of borderline Sts, one, one, and seven activities showed the largest total area under TCs 2, 0.3, and 0.1 s, respectively (Analysis B in Figure 4). Thus, nine borderline Sts showed a similar tendency to that of 27 Sts under three different TCs.

### High-Frequency Activity Analysis

**FIGURE 8:**
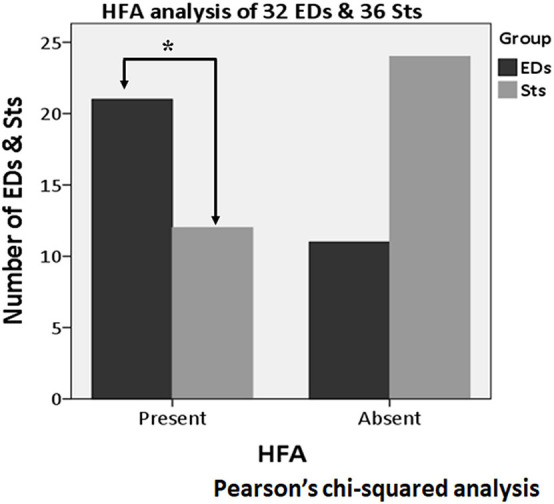
High-frequency activity (HFA) analysis of all 68 activities (32 EDs and 36 Sts). Compared to Sts (12 activities), HFA is found significantly more often with the apical component of EDs (21 activities) (*p* = 0.008). *Indicates statistical significance.

**FIGURE 9:**
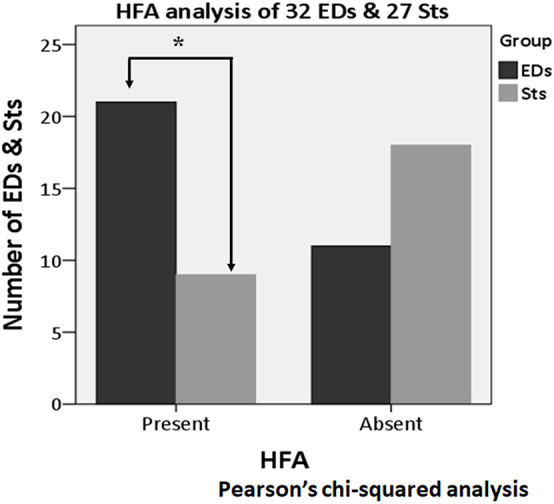
High-frequency activity analysis of 59 activities (32 EDs and 27 Sts) after excluding borderline nine Sts. HFA is significantly more associated with EDs (21 activities) as compared with Sts (nine activities) (*p* = 0.013). *Indicates statistical significance.

About the analysis of HFA, among all 68 activities (32 EDs and 36 Sts), HFA was found with the apical component of 21 EDs (65.6%) and 12 Sts (33.3%). In the Pearson’s chi-squared analysis of all 68 activities (32 EDs and 36 Sts), it was found that the HFA was significantly more often with the apical component of EDs as compared with Sts (*p* = 0.008) (Figure 8). Furthermore, after exclusion of borderline nine Sts (32 EDs and 27 Sts), HFA remains significantly more associated with EDs (21 activities, 65.6%) in comparison to Sts (nine activities, 33.3%) (*p* = 0.013) (Figure 9).

## Discussion

In this study, we demonstrated two main findings for the real world’s patient EEG data. First, the total area of the after-slow activity of EDs with long TC (2 s) was significantly larger than that of conventional (0.3 s) and short (0.1 s) TCs. By contrast, no significant difference in the same parameter of Sts was observed. Second, the total duration of the after-slow activity of EDs was also significantly longer than that of Sts.

### Change in the Total Area of the After-Slow Under Long Time Constant

To the best of our knowledge, this is the first systematic study to disclose that long TC is useful for defining EDs by the total area of the after-slow in comparison with Sts. Although EDs showed a significantly larger total area of the after-slow than that of Sts even with TC 0.3 s, our findings indicate that the enlarged total area in long TC is also a useful biomarker to differentiate EDs from Sts, especially when equivocal after-slow waves are observed by TC 0.3 or 0.1 s (Figures 5A,C). Our findings further support the IFCN guideline stating that the low filter should be set to 0.16 Hz or less for recording except for specific or difficult clinical conditions (Nuwer et al., 1998).

Regarding EDs recorded from scalp EEG as field potentials, the apical component reflects the synchronized intracellular PDS, i.e., giant, abnormal excitatory postsynaptic potentials from the generator point of view, mainly occurring in the cortical pyramidal neurons. They are immediately followed by hyperpolarized activity as the recurrent inhibition that corresponds to after-slow activity at least in the experimental data (Engel, 1989). Therefore, even in the clinical EEG, after-slow is also an important parameter for defining EDs. On the other hand, possible generator mechanisms of Sts depend on its definition as follows. Namely, it is noted that (a) very ill-defined EDs and (b) the pyramidal neurons in the cerebral cortices that generate physiological oscillations at frequencies similar to spikes (i.e., beta oscillations and mu rhythms), can be prominent in certain regions (Janca et al., 2014). For EDs, the total duration of after-slow was significantly longer than that of Sts as indicated in result section “Analysis of Total Area and Total Duration of After Slow with Time Constant of 0.3 s.” Compared to physiological EEG activity, the hyperpolarized activity immediately after PDS contains a slower frequency band because of prolonging depression in the excitability during this phase as described in previous human intracranial EEG studies (Neckelmann et al., 2000; Urrestarazu et al., 2006; Matsumoto et al., 2013; Von Ellenrieder et al., 2016). Based on these findings, our findings suggest that long TC may be useful for the selective enhancement of hyperpolarized activity (after-slow) immediately after PDS (EDs).

There may be some speculation that the augmentation of after-slow activity of EDs by changing TC setting is related to the longer total duration of apical component of EDs in addition to after-slow as described later. However, in this study, the total duration of the apical component did not correspond to the changes of the total area of after-slow in EDs and Sts (Supplementary Figures 1, 2).

### Total Duration of the After-Slow Under the Conventional Time Constant Condition (0.3 s)

Epileptiform discharges are defined to show clearer after-slow than Sts; therefore, the total duration was also significantly longer in EDs. The total duration may also be a useful parameter for differentiating after-slow activities of EDs from those of Sts.

### Analysis of Borderline Sharp Transients

With respect to the analysis of borderline nine Sts, the total area of after-slow activities showed a similar tendency to that of 27 Sts under three different TC conditions. It may imply that certified electroencephalographers visually inspected mainly the total area rather than the amplitude of after-slow activities that practically leads to the correct conclusion. Namely, although certified electroencephalographers explicitly understand the amplitude criteria, they implicitly make the judgment by the factor of total area. It may validate the total area as the important criteria to differ between EDs and Sts.

About the differences in the total area of the after-slow activity of all 36 Sts among three different TCs, although the total area of after-slow under TC 0.1 s (mean ± SD: 6.85 ± 5.37 μVs) was significantly larger than that under 2 s (6.60 ± 5.35 μVs) (*p* = 0.003), there were no significant differences between any other comparisons after Bonferroni adjustments. So, under long TC (2 s) setting the after-slow of Sts remain almost similar to that of under TC 0.3 s.

### High-Frequency Activity Analysis

High-frequency activity is considered to be associated with epileptogenicity (Shibata et al., 2016) because the increase of HFA is usually seen with the apical component of EDs (Kobayashi et al., 2009). As there is no definite characterization of HFA, the less strict inclusion criteria of two consecutive peaks were adopted in this study.

With respect to the other types of benign Sts such as benign focal EDs in childhood (Beaussart, 1972; Lüders et al., 1987) and benign epileptiform transients of sleep (White et al., 1977), these benign activities are usually accompanied by lower amplitude after-slow than that of the preceding apical component. To the best of our knowledge, as there is no clear definition or criteria of Sts with small after-slow except for anecdotal impression or experience like 50% of borderline, we initially selected the amplitude of after-slow more and less than 50% that of preceding apical component as the criteria of EDs and Sts, respectively. However, in this study, HFA analysis showed a clearer association with EDs than with Sts, which further justifies the differentiation of these two groups (EDs and Sts).

### Limitations

This study has some limitations. First, it was a retrospective study based on a relatively small number of EEG activities in a single institute. In addition, no specific patients were selected other than having epilepsy. Therefore, further studies with larger populations of patients from homogeneous clinical backgrounds would be warranted to confirm the present findings and to further clarify its clinical significance based on epilepsy classifications, namely, generalized and partial epilepsy. Second, we randomly chose relatively typical EDs and Sts waveforms, and also the number of each sample from each patient was relatively small. We purposely excluded pure spike without after-slow, since the pure spike is atypical except for repetitive spikes or polyspikes. Nevertheless, these waveforms are less frequent. Future studies involving these waveforms would also be necessary to clarify the utility in practical EEG settings. Third, we tentatively chose the amplitude of after-slow more and less than 50% that of the preceding apical component to differentiate EDs and Sts, respectively. Further studies in the clinical setting are needed to confirm the current findings and to define the criteria of EDs and Sts more precisely. Nevertheless, the consistent results using HFA could warrant the present finding. Fourth, in this study, we did not inspect possible mechanisms of neurons associated with different TC. Hence, future studies regarding these patterns of neuronal activity from the viewpoint of seizure would be useful to understand EDs further in the scalp EEG. Finally, long TC may provide or exaggerates not only after-slow activities, but also slower artifacts such as movements, sweat, slow eye movement, electrode lead wire movement, and so on. These slower artifacts may interfere with the interpretation of after-slow activities if their frequency is close to the frequency of after-slow.

Even though we take all of those limitations into account, the current study may shed the light on the useful approach of after-slow activity with a long TC of 2 s in scalp EEG to differentiate between EDs and Sts that was supported by HFA analysis.

## Conclusion

In this study, the EEG analysis showed that a long TC setting for EEG display is beneficial for differentiating after-slow activities between EDs and Sts. These findings are derived from the results of analysis on the total area of after-slow and supported by findings of HFA analysis. These findings at least may endorse the current operational definitions of EDs and Sts, and also the IFCN guidelines (1998). Total area rather than the amplitude of the after-slow was much more consistent with a visual inspection of EDs and Sts. Future studies to validate the clinical significance of current findings are, therefore, warranted.

## Meetings in Which the Study Has Been Presented

Preliminary results of this study were presented at 71st American Epilepsy Society Annual Meeting, Washington D.C., the United States of America, December 1–5, 2017; at XXIII World Congress of Neurology, Kyoto, Japan, September 16–21, 2017 and at the 50th Congress of the Japan Epilepsy Society, Shizuoka, Japan, October 7–9, 2016.

## Data Availability Statement

The raw data supporting the conclusions of this article will be made available by the authors, without undue reservation.

## Ethics Statement

The studies involving human participants were reviewed and approved by the institutional review board of Kyoto University (No. R0603). Written informed consent for participation was not required for this study in accordance with the national legislation and the institutional requirements.

## Author Contributions

SS did the initiation and planning of the project, did data collection and analysis, wrote the first draft of the manuscript, edited subsequent drafts, and created figures. TH aided in the planning of the project, verified the analytical methods, and edited the manuscripts. MD and AS provided additional patient information. MM contributed to the production of scripts for analyzing data from EEG. RT contributed to conception and design. AI contributed to the initiation and conceptualization of the project, supervised the findings of this study, and edited the manuscript. All authors contributed to the article and approved the submitted version.

## Conflict of Interest

AI and MM were members of the Department of Epilepsy, Movement Disorders and Physiology which is the Industry-Academia Collaboration Courses, supported by the Eisai Co., Ltd., Nihon Kohden Corporation, Otsuka Pharmaceutical Co., and UCB Japan Co., Ltd. The remaining authors declare that the research was conducted in the absence of any commercial or financial relationships that could be construed as a potential conflict of interest.

## Publisher’s Note

All claims expressed in this article are solely those of the authors and do not necessarily represent those of their affiliated organizations, or those of the publisher, the editors and the reviewers. Any product that may be evaluated in this article, or claim that may be made by its manufacturer, is not guaranteed or endorsed by the publisher.

## Supplementary Material

Supplementary Figure 1 Scatter plot with linear regression fit (solid line) and a 95% CI (dashed lines) for the correlation between the total area of the after-slow of all 32 EDs (26 sharp waves and six spikes) under three different TC conditions and total duration of the apical component under TC 0.3 s. No significant relation is observed between total duration of apical component under TC 0.3 s and total area of after-slow in **(A)** TCs 2 s (τb = 0.219), **(B)** 0.3 s (τb = 0.223), and **(C)** 0.1 s (τb = 0.235), respectively.

Supplementary Figure 2 The same graph is made for 27 Sts. No significant relation is found between the total duration of the apical component under TC 0.3 s and total area of after-slow in **(A)** TCs 2 s (τb = −0.191), **(B)** 0.3 s (τb = −0.129), and **(C)** 0.1 s (τb = −0.077), respectively.
