# Corrigendum: A Long Time Constant May Endorse Sharp Waves and Spikes Over Sharp Transients in Scalp Electroencephalography: A Comparison of After-Slow Among Different Time Constants Concordant With High-Frequency Activity Analysis

**DOI:** 10.3389/fnhum.2022.889046

**Published:** 2022-05-03

**Authors:** Shamima Sultana, Takefumi Hitomi, Masako Daifu Kobayashi, Akihiro Shimotake, Masao Matsuhashi, Ryosuke Takahashi, Akio Ikeda

**Affiliations:** ^1^Department of Neurology, Graduate School of Medicine, Kyoto University, Kyoto, Japan; ^2^Department of Clinical Laboratory Medicine, Graduate School of Medicine, Kyoto University, Kyoto, Japan; ^3^Department of Epilepsy, Movement Disorders and Physiology, Graduate School of Medicine, Kyoto University, Kyoto, Japan

**Keywords:** epileptiform discharge, total area, total duration, paroxysmal depolarization shifts, high-frequency activity

In the original article, there was an error. The title was mistakenly written as “Long Time Constant May Endorses Sharp Waves and Spikes Than Sharp Transients in Scalp Electroencephalography: A Comparison of Both After-Slow Among Different Time Constant and High-Frequency Activity Analysis.”

A correction has been made to the title, which now reads, “A Long Time Constant May Endorse Sharp Waves and Spikes Over Sharp Transients in Scalp Electroencephalography: A Comparison of After-Slow Among Different Time Constants Concordant With High-Frequency Activity Analysis.”

Additionally, there was a mistake in the citation for Masako Daifu Kobayashi. The correct citation is Diafu Kobayashi M.

The authors apologize for this error and state that this does not change the scientific conclusions of the article in any way. The original article has been updated.

## Publisher's Note

All claims expressed in this article are solely those of the authors and do not necessarily represent those of their affiliated organizations, or those of the publisher, the editors and the reviewers. Any product that may be evaluated in this article, or claim that may be made by its manufacturer, is not guaranteed or endorsed by the publisher.

