# Supplementary figures and images for: A Long Time Constant May Endorse Sharp Waves and Spikes Over Sharp Transients in Scalp Electroencephalography: A Comparison of After-Slow Among Different Time Constants Concordant With High-Frequency Activity Analysis
Source: Front Hum Neurosci. 2021 Oct 22;15:748893. doi: 10.3389/fnhum.2021.748893 (PMC8569184; doi:10.3389/fnhum.2021.748893)

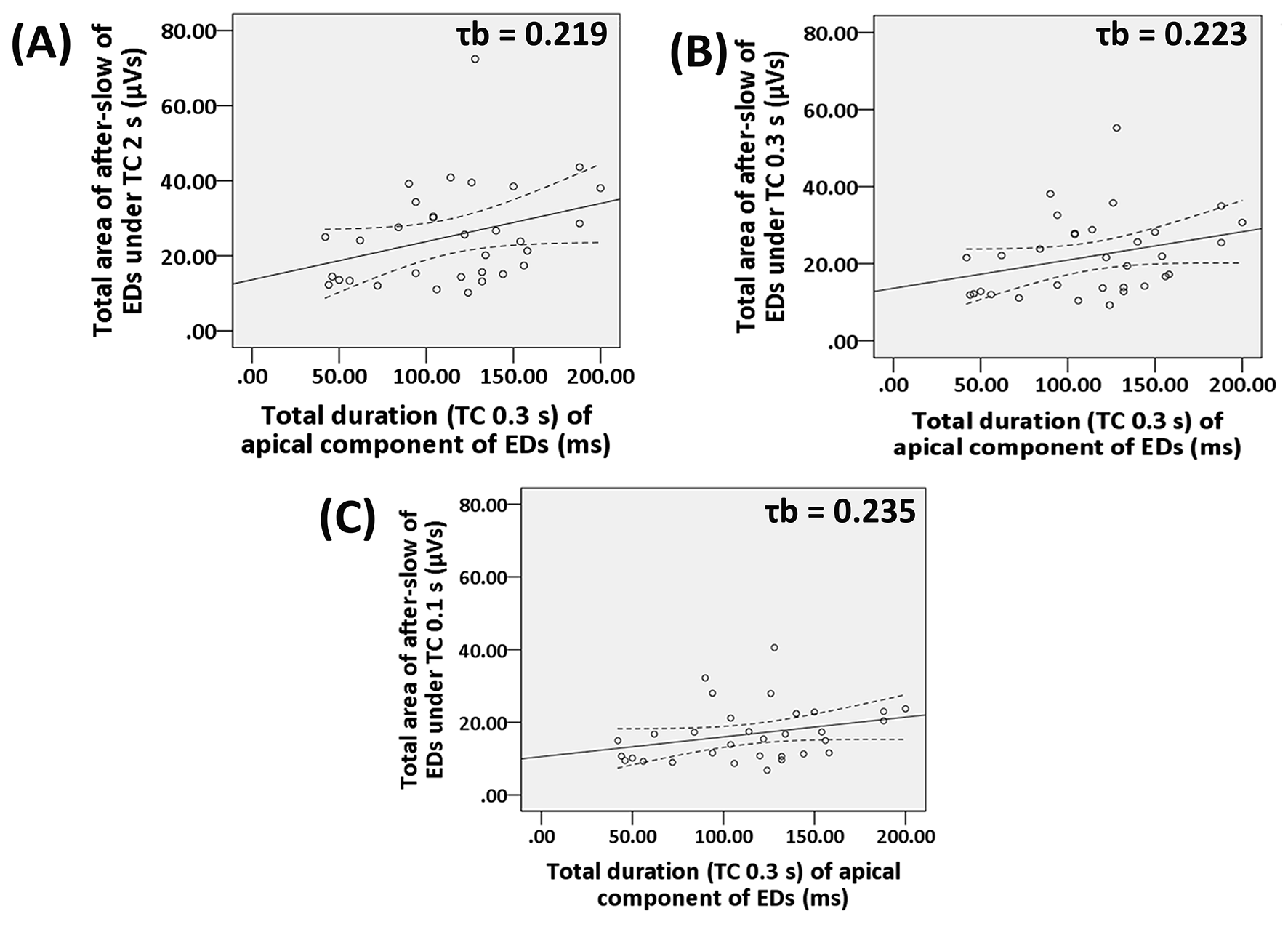

Supplement: Supplementary Figure 1 — Scatter plot with linear regression fit (solid line) and a 95% CI (dashed lines) for the correlation between the total area of the after-slow of all 32 EDs (26 sharp waves and six spikes) under three different TC conditions and total duration of the apical component under TC 0.3 s. No significant relation is observed between total duration of apical component under TC 0.3 s and total area of after-slow in (A) TCs 2 s (τb = 0.219), (B) 0.3 s (τb = 0.223), and (C) 0.1 s (τb = 0.235), respectively. [file Image_1.TIF]

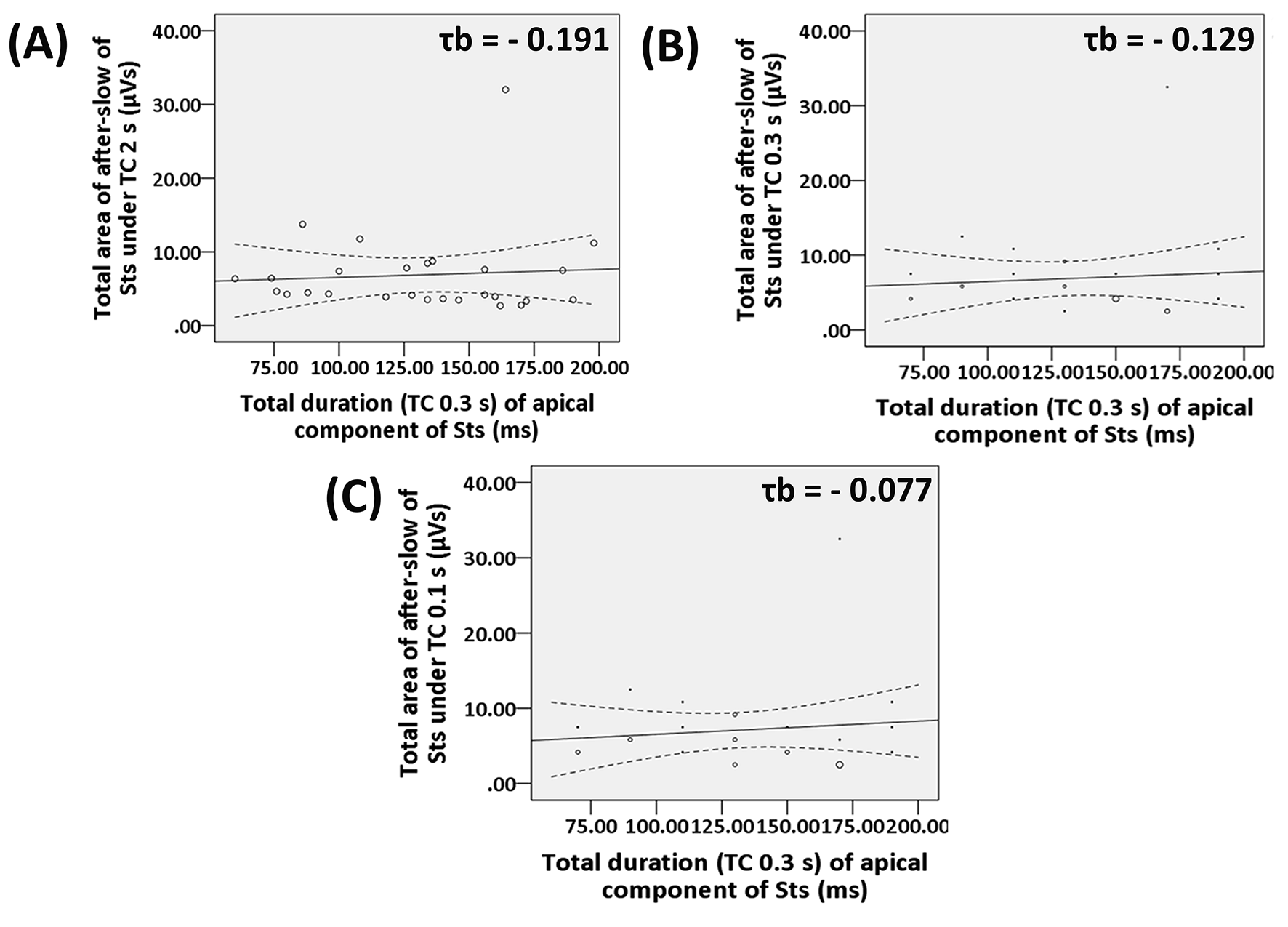

Supplement: Supplementary Figure 2 — The same graph is made for 27 Sts. No significant relation is found between the total duration of the apical component under TC 0.3 s and total area of after-slow in (A) TCs 2 s (τb = −0.191), (B) 0.3 s (τb = −0.129), and (C) 0.1 s (τb = −0.077), respectively. [file Image_2.TIF]
